# Supplementary material for: Stabilization of cultural innovations depends on population density: Testing an epidemiological model of cultural evolution against a global dataset of rock art sites and climate-based estimates of ancient population densities
Source: PLoS One. 2021 Mar 17;16(3):e0247973. doi: 10.1371/journal.pone.0247973 (PMC7968670; doi:10.1371/journal.pone.0247973)
Supplement: S1 Appendix — (PDF) [file pone.0247973.s001.pdf]

S1 Appendix

Derivation of the epidemiological model

The model describes the diffusion of a cultural innovation through a Culturally Effective Population (CEP) [12] i.e. a closed network of communicating individuals or subpopulations. Notation for the "disease dynamics" follows the conventions established in [29].

Consider the emergence of rock art in a CEP comprising N subpopulations or communities, where I and S are respectively the proportion of *infected* communities (communities that have adopted the innovation), and *susceptible* communities (communities that have not). If the population is "well-mixed" (i.e., every subpopulation is in social contact with every other subpopulation), any infected subpopulation can transmit the innovation to any susceptible subpopulation.

Let β be the rate of transmission per time step and γ the rate of recovery of the infected subpopulation (here: the rate at which it loses the ability to produce rock art). In these conditions, the rate of change in the proportion of infected subpopulations is given by:

$$\frac{dI}{dt} = \beta IS - \gamma I \qquad (1)$$

In a partially connected population, infected subpopulations can only transmit an innovation to the susceptible subpopulations with which it is in contact. Assuming each subpopulation is in contact with kS susceptible sub-populations, and substituting S with $1 - I$, we obtain

$$\frac{dI}{dt} = \beta kI(1 - I) - \gamma I \qquad (2)$$

Rearranging, solving for $\frac{dI}{dt} = 0$, and imposing $I^* \geq 0$, we obtain the stable proportion of infected subpopulations:

$$I^* = \begin{cases} 0,\ 1 - \frac{\gamma}{k\beta} \leq 0 \\ \left(1 - \frac{\lambda}{k\beta}\right), 1 - \frac{\gamma}{k\beta} > 0 \end{cases} \quad (3)$$

If rates of encounter between communities inhabiting a territory are proportional to the square root of the population density (i.e. k=$\alpha\sqrt{\rho}$):

$$\mathrm{I}^* = \begin{cases} 0,1 - \frac{\gamma}{\alpha\beta\sqrt{\rho}} \leq 0 \\ 1 - \frac{\gamma}{\alpha\beta\sqrt{\rho}}, 1 - \frac{\gamma}{\alpha\beta\sqrt{\rho}} > 0 \end{cases} \quad (4)$$

At ρ^*, $1 - \frac{\gamma}{\alpha\beta\sqrt{p}} = 0$. Thus:

$$\rho^* = \left(\frac{\gamma}{\alpha\beta}\right)^2 \quad (5)$$

To reduce the number of parameters in the model, we normalize the rate of transmission, setting $\alpha\beta$ to 1. We thus obtain the simplified expressions:

$$\rho^* = \gamma^2 \quad (6)$$

and

$$I^* = \begin{cases} 0, \rho \leq \rho^* \\ 1 - \sqrt{\frac{\rho^*}{\rho}}, \rho > \rho^* \end{cases} \quad (7)$$

To test our model, we consider the empirical *site detection ratio, P,* as defined in the main text.

Assuming that each infected subpopulation has an equal probability, z, of producing an artifact that produces a trace in the archaeological record, the probability *P,* that *N* infected subpopulations will generate at least one such trace, is given by:

$$P = (1-(1 - zI^*)^N) \quad (8)$$

Assuming *z* is small,

$$P \cong zI^*N \tag{9}$$

The value of *N* for a cell of standard area, with fixed mean community size, is proportional to the population density for the cell:

$$N = \mu\rho \tag{10}$$

Grouping μ and z in a single variable ζ we obtain

$$P \cong \zeta\, I^*\rho \tag{11}$$

As described in the main text, an error term ε represents the probability that a site is attributed to a cell with an incorrect population. Thus:

$$P = (1 - \varepsilon)\zeta I^*\rho + \varepsilon \tag{12}$$

This is the model we tested in our empirical study.
