# Supplementary material for: Stabilization of cultural innovations depends on population density: Testing an epidemiological model of cultural evolution against a global dataset of rock art sites and climate-based estimates of ancient population densities
Source: PLoS One. 2021 Mar 17;16(3):e0247973. doi: 10.1371/journal.pone.0247973 (PMC7968670; doi:10.1371/journal.pone.0247973)
Supplement: S1 Table — (PDF) [file pone.0247973.s002.pdf]

S1 Table
Rock art dataset (133 sites)

Name	Latitude	Longitude	Earliest age in sample	Latest age in sample	Modern Country	Date of reference	Dating method/comments	Direct / indirect	Exact Age / Minimum Age / Max Age	Calibrated	Kind	Figurative	Reference
Abri Castanet, Dordogne, France	44.999272	1.101261	37'205	36'385	France	2012	accelerator mass spectrometry (AMS), indirect (bone samples)	Indirect	Minimum Age	Yes	Petroglyphs	Yes	(1)
Altamira, Spain	43.377452	-4.122347	36'160	2'850	Spain	2013	Uranium-series dating	Direct	Exact Age	N/A	Petroglyphs Decorated ceiling in cave	Yes, not all. One is linear red line.	(2)
Altxerri B, Spain	43.2369	-2.148555	39'479	34'689	Spain	2013	Indirect radiocarbon dating	Indirect	Minimum age	Yes	Painting	Yes	(3)
Anbarndarr I. Australia/Anbarndarr II, Australia/Gunbirdi I, Gunbirdi II, Gunbirdi III, Northern Territory Australia	-12.255207	133.645845	1'704	111	Australia	2010	Direct radiocarbon dating of beeswax sample	Direct	Exact age	Yes	Beeswax	No	(4)
Anta de Serramo, Vimianzo, A Coruña, Galicia, Spain	43.110048	-9.03242	6'950	6'950	Spain	2005	Direct radiocarbon dating, by AMS on charcoal	Direct	Exact age	Yes	Painting	N/A	(5)
Apollo 11 Cave, IlKaras Region, Southwest Namibia	-26.842964	17.290284	32332*	30654*	Namibia	1983	Radiocarbon dating	Indirect	Minimum age	Unknown	Painted fragments	Yes	(6)
ARN-0063, Namarrgon Lightning Man, Northern Territory, Australia	-12.865524	132.814001	1'021	145	Australia	2010	Direct radiocarbon from beeswax	Direct	Exact age	Yes	Beeswax	Yes	(4)
Bald Rock, Wellington Range,Northern Territory Australia	-11.8	133.15	386	174	Australia	2010	Direct radiocarbon from beeswax	Direct	Exact age	Yes	Beeswax	N/A	(4)
Baroalba Springs, Kakadu, Northern Territory, Australia	-12.677013	132.480901	7'876	7'876	Australia	2010	Indirect radiocarbon dating via minimum age of mineral salts from rock art surface sampled	Indirect	Minimum age	Yes	Painting	-	(4)
Bhimbetka, India	22.939546	77.612433	5953*	1107*	India	2005	Direct radiocarbon dating on white paint from figure	Direct	Exact Age	Unknown	Painting	Yes	(7)
Biggarsberg, South Africa	-26.286668	28.047223	4'050	970	South Africa	2003	Direct radiocarbon from oxalates underlying and overlying painting	Direct	Exact age	Yes	Painting	N/A	(8)
Bighorn Basin, Wyoming and Montana, USA	44.379957	-108.038989	6'862	0	USA	1993	AMS Radiocarbon dating	Direct	Exact age	Yes	Petroglyphs	N/A	(9)
Billasurgam, Kurnool, Andra Panesh, India	15.828126	78.037279	5'620	4'825	India	2012	Radiocarbon dating of flowstone on top and below the engraving	Indirect	Minimum Age	Yes	Petroglyph	No	(10)
Bindu, Kakadu, Northern Territory, Australia	-12.677013	132.480901	183	117	Australia	2010	Direct radiocarbon from beeswax	Direct	Exact age	Yes	Beeswax dots and lines	No	(4)
Bush Turkey Dreaming Site, Western Australia	-23.96	122.97	60	60	Australia	2010	Direct radiocarbon from beeswax	Direct	Exact age	Yes	Painting	Yes	(4)
Cape York, Australia	-10.7	142.516667	678*	675*	Australia	1993	Direct radiocarbon dating on plant-fibre binders	Direct	Exact age	Unknown	Painting	N/A	(11)
Casota do Paramo, A Coruña, Spain	42.704644	-8.930863	5'400	5'400	Spain	2005	Direct radiocarbon dating of charcoal paint samples	Direct	Maximum Age	Yes	Painting	No	(5)
Cave of Beasts, Gilf Kebir, Egypt	23.441389	25.839722	8'450	6'350	Egypt	2014	Radiocarbon dating, pottery typology	Indirect	N/A	N/A	Painting	Yes	(12)
Cave of Bees, Matopos, Zimbabwe	-20.557222	28.5125	12`462*	12'462*	South Africa	1983	Radiocarbon dating on painted spall	Direct	Exact age	Unknown	Painting	N/A	(6)
Cave of Cougnac, France	44.756986	1.375957	28'007*	28'007*	France	2001	Direct radiocarbon dating on charcoal from paintings	Direct	Exact Age	Unknown	Painting	Yes (2 deers)	(13)
Cave of El Castillo, Spain	43.292368	-3.965576	41'400	22'880	Spain	2012, 2001,	Uranium Series disequilibrium dates of calcite deposits overlying or underlying art	Direct	Minimum Age	N/A	Pike: Painting	Pike: Some, most are disk-shapes Valladas 2001: Yes Valladas 1992: Yes	(14)
Cave of Niaux, France	42.820098	1.593487	15,390*	15390*	France	1992	Valladas 1992: Direct radiocarbon dating Clottes 1992:	Direct	Exact age	Unknown	Painting	Yes	(15)
Chauvet-Pont-d'Arc Cave, France	44.387244	4.415865	36292*	34337*	France	2001	Direct radiocarbon dating from painting	Direct	Exact age	Unknown.	Painting	Yes	(16)
Chifubwa Stream Shelter, Zambia	-12.2	26.683333	7227*	7227*	Zambia	1979	Indirect radiocarbon	Indirect	Minimum age	Unknown	Engraving	No	(17)
Coliboaia Cave, Romania	46.53089	22.597332	35'917	35'917*	Romania	2012	Radiocarbon dating of charcoal layer atop drawing	Direct	Exact age	Unknown	Drawings	Yes	(18)
Cosquer cave, Calanque de Morgiou, France	43.210931	5.445896	32'190*	21'567*	France	2001	Radiocarbon dating	Direct	Exact Age	No	Painting	Yes	(16)
Coto dos Muros, Baixo Miño, Northwest Iberia	41.999517	-8.770147	6'340	4'020	Spain	2005	Radiocarbon dating	Direct	Maximum Age	Yes	Painting	N/A	(5)
Covaciella, Spain	43.318232	-4.875262	17113	16'881	Spain	2001	Radiocarbon dating	Yes	Exact age	Yes	Painting	Yes	(16)
Deighton Lady, Jowalbinna, Queensland Australia	-15.5333	144.7833	6'483	2'905	Australia	2010	Minimum age radiocarbon dating based on minimum age of oxalate sample	Indirect	Minimum age	Yes	Engraving	No	(4)
Djarrng, West Arnhem Land, Northern Territory, Australia	-12.091834	132.890495	720	119	Australia	2010	Direct radiocarbon from beeswax	Direct	Exact age	Yes	Beeswax	Yes	(4)
Djulirri, Arnhem Land, Northerm Territory, Australia	-12.091834	132.890495	383	172	Australia	2010	Direct radiocarbon from beeswax	Direct	Exact age	Yes	Beeswax	Yes	(4)
East Alligator River, Northern Territory, Australia	-12.525429	133.060161	4'287	122	Australia	2010	Direct radiocarbon from beeswax	Direct	Exact age	Yes	Beeswax	No	(4)
Echidna's Rest, Queensland, Australia	-27.542345	153.07315	1'291	Modern	Australia	2010	Direct radiocarbon from charcoal	Direct	Exact age	Yes	Drawings and engraving	No	(4)
El Hosh, Egypt (6km north of Gebel Silsila)	24.642614	32.92968	7'550	2'325	Egypt	2001	Direct radiocarbon dating	Direct	Exact Age	Yes	Petroglyphs	Yes	(19)
Emu Cave, New South Wales, Australia	-33.782342	150.590426	1'857	1'857	Australia	2010	Radiocarboon dating on sample collected from over an emu track	Indirect	Minimum age	Yes	Engraving	Yes	(4)
Fariseu, Coa Valley, Portugal	41.079991	-7.111782	18'400	10'800	Portugal	2006	optical luminescence (OSL) from burnt lithic remains	Indirect	Minimum age	N/A	Petroglyphs	Yes	(20)
Fetra Hé Cave, Lifou Island, New Caledonia	-21.038497	167.240515	2'570	983	New Caledonia	2006	Radiocarbon dating	Direct	Exact age	Yes	Hand stencils	Some	(21)
Forno dos muros, portugal	40.224238	-8.636221	5'840	5'505	Portugal	2005	Direct radiocarbon	Direct	Exact age	Yes	Painting	N/A	(5)
Foz Coa, Portugal	41.082247	-7.14036	2'000	100	Portugal	1996	Radiocarbon dating of microorganisms in cracks in engravings	Indirect	Minimum age	N/A	Petroglyphs	Yes	(22)
Fuente de el Salin, Muñorrodero Spain	43.364429	-4.482981	26'595*	26'596*	Spain	2017	Indirect radiocarbon dating on charcoal from associated hearth	Indirect	Minimum age	Unknown	Painting	N/A	(23)
Gargas, France	43.90322	5.357388	30'988*	30'988*	France	1992	Radiocarbon dating	Indirect	Minimum age	Unknown	Hand stencils, engravings	Yes	(24)
Giant Horse, Laura, Queensland, Australia	-15.5384	144.4566	474	474	Australia	2010	Direct radiocarbon in layered paint	Direct	Exact age	Yes	Painting	-	(4)

Glen Canyon, South-central Utah, USA	37.386989	-110.842823	1'080	675	USA	1992	Radiocarbon dating of associated artifacts	Indirect	N/A	Unknown	Painting	Yes	(25)
Grande Grotte, Arcy-sur-Cure, France	47.591452	3.766392	45'504	29'173	France	2013	Indirect radiocarbon dating on associated bone and tooth specimens	Indirect	N/A	Yes	Painting	N/A	(26)
Grotta di Fumane, Italy	45.592481	10.90443	41'000	41'000	Italy	2009	Radiocarbon dating of wood carbon associated with paintings	Indirect	Exact Age	Yes	Painting	Yes	(27)
Grotte d'Aldène, Cesseras, France	43.353897	2.698387	34'263*	34'263*	France	2005	AMS	Indirect	N/A	Unknown	Engravings	N/A	(28)
Grotte de Cussac, Dordogne River Valley, France	44.829444	0.847778	29'209	3'426	France	2017	U eTh and 14C for speleothems, 14C-AMS for organic materials, bone and charcoal	Indirect	Mixed	Unknown	Painting	Yes	(29)
Gua Saleh, Sangkuliran - Mangkalihat Peninsula, Kalimantan Island, Indonesia	1.035717	118.065445	27'270	9'820	Indonesia	2003	Cross-dating using Th/U and AMS of calcite covering paintings	Indirect	Minimum age	Yes	Paintings	Yes	(30)
Gunbilngmurrung, Australia	-12.8	133.3	4'297*	145*	Australia	2010	Direct radiocarbon on beeswax	Direct	Exact Age	Yes	Beeswax	Yes	(31)
Hathitol, India	23.152243	79.920672	5'526*	2'882*	India	2005	DIrect radiocarbon on painting pigment	Direct	Exact Age	Unknown	Painting	Yes	(7)
Hay Cave, Queensland, Australia	-23.167328	150.490387	2'484*	925*	Australia	2010	Direct charcoal pigr	Direct	Exact age	No	Painting	No	(4)
Ignatievskaya Cave, Russia	54.899497	57.781586	8790	6925	Russia	2002	Radiocarbon dating on charcoal pigment	Direct	Exact Age	Yes	Painting	Yes	(32)
Ingaladdi, willeroo, Northern Territory, Australia	-15.29028	131.575195	7'676	7'676	Australia	2010	Radiocarbon dating on charcoal sampled using minimum age	Indirect	Minimum age	Yes	Engraving	N/A	(4)
Jamjori, India	24.515475	87.081156	1'634*	1'634*	India	2005	Direct radiocarbon dating on paint	Direct	Exact Age	Unknown	Painting	No	(7)
Jinsha, China	26.855047	100.22775	5'216	5'216	China	2012	Uranium-series dating on calcite samples above and beneath painting	Indirect	Maximum Age	N/A	Painting	Yes	(33)
Kaalpi site, Western Australia (Near Calvert Ranges)	-23.95	122.72	377	308	Australia	2010	Radiocarbon dating of sample pigment	Direct	Exact age	Yes	Painting	N/A	(4)
Kaho'olawe, Hawai'i, USA	20.558047	-156.605738	923	0	Hawaii	1996	Radiocarbon dating of sampled petroglyph	Indirect	Minimum age	Yes	Painting	Yes	(34)
Kapova cave, Russia	53.043799	57.065139	17,870	16'893	Russia	1989	Radiocarbon dating of charcoal from cultural stratum	Indirect	Unknown	Unknown	Painting	Yes	(35).
Kennedy River, Queensland, Australia	-15.5906	144.0308	1'132	1'132	Australia	2010	Radiocarbon dating of oxalate sample	Indirect	Minimum age	Yes	Engraving	Yes	(4)
Kimberley, Australia	-17.34918	125.915207	4'300	1'350	Australia	2010	Radiocarbon dating of paint	Direct	Exact age	Yes	Painting	Yes	(4)
La Garma, Cantabria, Spain	43.151944	-3.896389	16'875	16'875	Spain	2013	Direct radiocarbon dating	Direct	Exact age	Yes	Paintings	Yes	(26)
La Pileta, Spain	36.691297	-5.2699	24'228	24'228	Spain	2013	Direct radiocarbon dating	Direct	Exact age	Yes	Paintings	Yes	(26)
La Tête du lion, France	45.777403	4.855214	26'760	25'997	France	2013	Indirect radiocarbon dating on charcoal	Indirect	Minimum age	Yes	Paintings	N/A	(26)
Labastide, France	43.035546	0.352267	17'476	484	France	2013	Radiocarbon dating	Direct	Exact age	Yes	Paintings	Yes	(26)
Lapa do Santo, Brazil	-19.477778	-44.038889	10'600	10'600	Brazil	2012	Direct radiocarbon ages assisted by OSL	Yes	Exact age	Yes	Petroglyph	Yes	(36)
Lascaux Cave, France	45.053919	1.167651	22'723	18'848	France	2013	Radiocarbon dating	Indirect	Minimum age	Yes	Paintings	Yes	(26)
Lene Hara Cave, East Timor	-8.411695	127.293235	11'350	11'350	East Timor	2007	Plasma mass spectrometry	Direct	Exact age	Yes	Paintings	N/A	(37)
Lower Pecos, Texas	31.422912	-103.493229	4'290*	4'290*	USA	1995	Direct AMS C14	Direct	Exact Age	Unknown	Painting	N/A	(38)
M23, Calvert Ranges, Western Australia	-23.966667	122.966667	2'085	759	Australia	2010	Direct radiocarbon dating of pigment sampled	Direct	Exact age	Yes	Painting	N/A	(4)
Malangine Cave, Nene Valley, South Australia	-38.05	140.69	6'348	5'046	Australia	2010	Radiocarbon dating of speleothem deposit	Indirect	Minimum age	Yes	Figure Fluting & Engraving	No	(4)
Mámoa do Monte: Dos Marxos	42.648475	-7.946347	6'110	5'495	Portugal	2005	Direct AMS C14 of paint	Direct	Exact age	Yes	Painting	N/A	(5)
Marjar - East Alligator, Northern territory, Australia	-13.092293	132.393766	191	191	Australia	2010	Direct radiocarbon dating of beeswax sample from motif	Direct	Exact age	Yes	Beeswax	N/A	(4)
Mennge-ya 1, Northern Territory (near Willeroo)	-15.29028	131.575195	4'488*	4'488*	Australia	2010	Radiocarbon dating of base of the cortex	Indirect	Minimum age	No	Engraving	No	(4)
Messak Plateau, Fezzan, Libya	25.75	11.833333	6'946*	5'906*	Libya	2010	Radiocarbon dating on associated stone monuments	Indirect	N/A	Unknown	Engraving	N/A	(39)
Mickey Springs 31, Hughenden, Queensland, Australia	-20.85	144.2	5'929	5'929	Australia	2010	Radiocarbon dating	Unknown	Unknown	Yes	Engraving	No	(4)
Moonface site, Queensland, Australia	-21.5097	140.6679	796	796	Australia	2010	Direct radiocarbon dating of charcoal sampled	Direct	Exact age	Yes	Painting	Yes	(4)
Moses Cave, Arnhem Land, Northern Territory, Australia	-12.430204	130.882379	274	274	Australia	2010	Direct radiocarbon from beeswax sample from motif	Direct	Exact age	Yes	Beeswax	No	(4)
Mount Manning, Western Australia	-30.12203	119.742604	586	182	Australia	2010	Indirect radiocarbon dating of charcoal ochre layers associated with drawings	Indirect	N/A	Yes	Drawing	No	(4)
Mungana Site, Queensland, Australia	-17.118416	144.398988	3'644	1'177	Australia	2010	Direct radiocarbon dating of charcoal sampled	Direct	Exact age	Yes	Drawing	No	(4)
Nangalor (Nanguluwur), Kakadu National Park, Northern Territory, Australia	-13.092293	132.393766	5'570	776	Australia	2010	Indirect radiocarbon dating of mineral salts from rock art surface sampled	Indirect	Minimum Age	Yes	Painting	No	(4)
Nara Inlet, Queensland	-20.15°	148.9	2'489	2'489	Australia	2010	Radiocarbon dating of charcoal sample	Unknown	Unknown	Yes	Unknown	N/A	(4)
Natal Drakkenberg, South Africa	-29.466667	29.266667	675	402	South Africa	1997	AMS Radiocarbon dating Andra 3 through associated plant fibers	Indirect	N/A	Yes	Painting	No	(40)
Nawarla Gabarnmang, Jawoyn Country, Arnhem Land Australia	-12.1685	133.8335	27630	27630	Australia	2013	Direct radiocarbon dating of charcoal pigment	Direct	Exact age	Yes	Painting	N/A	(41)
Nerja, Spain	36.785916	-3.804483	23'821	23'821	Spain	2013	Radiocarbon dating	Indirect	Minimum age	Yes	Drawings and torch rubbings	N/A	(26)

Ngarradj Warde Djobkeng, Northern Territory	-12.3	132.57	10'083	9'171	Australia	2010	Indirect radiocarbon dating of mineral salts from rock art surface sampled	Indirect	Minimum age	Yes	Painting	N/A	(4)
Ojo Guareña, Burgos, Spain	43.034337	-3.66452	13'377*	12'806*	Spain	2009	Direct radiocarbon dating of coal pigment	Direct	Exact age	Unknown	Paintings	Yes	(42)
Padahlin, Taunggyi District, Shan State, Myanmar	20.763697	96.920913	8'515*	1'664*	Myanmar	1971	AMS Radiocarbon dating	Indirect	Unknown	Unknown	Painting	Yes	(43)
Painted Shelter, Queensland, Australia	-16.1295	144.1289	2484*	2484*	Australia	2010	Direct radiocarbon	Direct	Exact Age	No	Painting	Yes	(4)
Panaramitee, Flinders Ranges, South Australia	-31.416667	138.75	46'273	3'876	Australia	2010	Indirect radiocarbon dating of rock varnish	Indirect	Minimum age	No	Engraving	No	(4)
Pech Merle, France	44.507308	1.64408	28'727*	28'727*	France	2001	Radiocarbon dating	Direct	Exact age	Unknown	Painting	Yes	(13)
Pedra Cuberta	43.089554	-8.984605	5'775	5'620	Spain	2005	Radiocarbon AMS	Direct	Exact	Yes	Painting	Yes	(5)
Pedra da Moura, Vimianzo, A Coruña, Spain	43.081351	-8.977859	5'745	5'745	Spain	2005	Direct radiocarbon dating from megalithic paint	Direct	Exact age	Yes	Painting	N/A	(5)
Pedra Pintada, Monte Alegre, Brazil	-2.051761	54.182912	13'019*	11'950*	Brazil	1996	Radiocarbon dating (56) and thermoluminescen ce (13)	Indirect	Minimum age	Unknown	Painting	Yes	(44)
Pete's Chase, Queensland	-16.1295	144.1289	2'064	385	Australia	2010	Direct radiocarbon dating of pigment sampled	Direct	Exact age	No	Painting	Yes	(4)
Pilbara, Western Australia, Australia	-21.883333	116.766667	4'040*	2'731*	Australia	2009	Radiocarbon dating	Indirect	Minimum age	Unknown	Petroglyphs	Yes	(45)
Pomongwe Cave, Zimbabwe	-20.547412°	28.513674	5'526*	4'611*	Zimbabwe	1983	Direct radiocarbon dating from spalls with paint from 2 layers	Direct	Exact age	Unknown	Painting	N/A	(6)
Pondra, Cantabria, Spain	43.266475	-3.423179	22'000	22'000	Spain	2013	Thermoluminescen	Indirect	Minimum age	N/A	Painting	Yes	(46)
Possum Cave, Queensland, Australia	-10.7°	142.516667	9'501	2'217	Australia	2010	Radiocarbon dating on oxalate sampled	Indirect	Minimum age	Yes	Engraving	No	(4)
Prung-Cart Cave, South Australia (near Millicent)	-37.090413	140.829791	2'783	1'076	Australia	2010	Radiocarbon dating on calcite sampled	Indirect	Minimum age	Yes	Figure fluting	Unknown	(4)
Puritijarra, Cleland Hills, Northern Territory, Australia	-23.883333	130.866667	2'747	548	Australia	2010	Radiocarbon dating on oxalate sampled	Indirect	Minimum age	Yes	Engraving	No	(4)
Quinkans B6 Rockshelter, Queensland, Australia	-15.6286	144.5308	2'999	845	Australia	2010	Radiocarbon dating on oxalate sampled	Indirect	Minimum Age	Yes	Engraving & Painting	No	(4)
Qurta, Egypt	24.629167	32.9625	17'000	10'000	Egypt	2011	Direct Optically Stimulated Luminescence (OSL)	Direct	Exact Age	N/A	Petroglyph	Yes	(47)
Racecourse Site, Queensland, Australia	-17.1747	144.4994	2'031	2'031	Australia	2010	Direct radiocarbon dating on charcoal pigment	Direct	Exact Age	Yes	Drawing	No	(4)
Red Lady, Queensland, Australia	-15.762116	144.257562	8'213	8'213	Australia	2010	Indirect radiocarbon dating on silica sampled	Indirect	Minimum Age	Yes	Painting	Yes	(4)
Robin Hood Cave and Church Hole, Creswell Crags, UK	53.263491	-1.193529	15'700	13'200	UK	2005	Uranium-series disequilibrium dating on thin layer of flowstones covering the surface (Minimum age)	Indirect	Minimum Age	Yes	Engravings	Yes	(48)
RSA TYN2 , Drakensberg Mountains, Eastern Cape, South Africa	-29.466667	29.266667	2'009	1'971	South Africa	2011	Direct radiocarbon dating	Direct	Exact age	Yes	Paintings	Unknown	(49)
Sandy Creek 1 & 2, Queensland, Australia	-16	144	33'676*	3'126	Australia	2005	Indirect radiocarbon dating on oxalate sampled	Indirect	Minimum Age	Yes	Painting	Yes	(50)
Serpent's Glen 1, Wiluna, Western Australia	-25.216667	120.75	3'417	377	Australia	2010	Direct radiocarbon dating on paint sampled	Direct	Exact Age	Yes	Painting	No	(4)
Serra de Capivara, Brazil	-8.695278	-42.586267	9'863	1'028	Brazil	2013	Radiocarbon dating	Indirect	N/A	Yes	Paintings	N/A	(51)
Snake Site, Cannon Hill, Northern Territory, Australia	-12.3	132.57	563	563	Australia	2010	Indirect radiocarbon dating on mineral salts from rock art surface	Indirect	Minimum age	Yes	Painting	N/A	(4)
Spirit Cave, Anbangbang, Kakadu, Northern Territory, Australia	-12.865524	132.814001	14'182	3'747	Australia	2010	Indirect radiocarbon dating on oxalate sampled	Indirect	Minimum Age	Yes	Painting	No	(4)
Split rock, Queensland, Australia	-15.6525	144.4975	7'433	7'433	Australia	2010	Direct radiocarbon dating on silica sampled	Direct	Exact Age	Yes	Painting	N/A	(4)
Steenbokfontein Cave, South Africa	-32.161667	18.333333	9'392*	2'220*	South Africa	1999	AMS dating	Indirect	Minimum age	Unknown	Painting	No	(52)
Sturt Meadows, New South Wales, Australia	-31.11	140.4	12'237	11'978	Australia	2010	Indirect radiocarbon dating on calcium carbonate sampled	Indirect	Minimum Age	Yes	Engraving	N/A	(4)
Sulawesi, Indonesia	-1.8479	120.5279	44'000	17'770	Indonesia	2014	Uranium-series dating of coralloid speleothems	Indirect	Minimum age	Yes	Hand stencils and paintings	Yes	(53)
Tassili-n-Ajjer, Algeria	25.813595	8.133856	8'237	2'287	Algeria	2012	AMS Radiocarbon dating	Indirect	N/A	Yes	Paintings	Yes	(54)
Tennessee, Cumberland Plateau	35.949603	-85.027047	5'698	N/A	USA	2013	AMS Radiocarbon dating	Direct	Exact Age	Yes	Charcoal pictograph	Yes	(55)
Tito Bustillo Cave, Spain	43.460706	-5.067392	36'200	29'650	Spain	2012	U-series disequilibrium dating	Indirect	Mixed	Yes	Paintings	Yes	(14)
uKhalamba, South Africa	-29.380485	29.546	4'050	990	South Africa	2003	Indirect radiocarbo	Indirect	Maximum age	Yes	Painting	Yes	(8)
Unnamed sites 1-3, Kimberley, Western Australia	-17.34918	125.915207	23'800	Modern	Australia	2010	Indirect radiocarbon dating from sampled mud-wasp nest overlying paintings	Indirect	Minimum Age	No	Painting	Yes	(4)
Urdiales, Spain	43.368822	-3.215635	15'223	15'223	Spain	2013	Radiocarbon dating	Direct	Exact age	Yes	Painting	Yes	(26)
Urkan-e-Rub, Lower Jordan Valley, Israel	32.422877	35.302723	20'204*	20'204*	Israel	2010	Unknown	Unknown	Unknown	Unknown	Engraving	No	(56)
Villars, France	45.442277	0.785135	21'735	17'473	France	2013	Radiocarbon dating	Direct	Exact age	Yes	Drawings and torch rubbings	No	(26)
Walkunder Arch Cave, Queensland, Australia	-17.2297	144.5169	34'254	3'575	Australia	2010	Indirect radiocarbon dating on oxalate, graphite sampled	Indirect	Maximum age for date to use; different samples have minimum age, maximum age, middle date, and supporting date	Yes	Paintings & Engraving	N/A	(4)
Wanga East, Northern Territory, Australia	-24.19	131.4	8'093	1'332	Australia	2010	Indirect radiocarbon dating on oxalate formation	Indirect	Minimum age	Yes	Engraving	Yes	(4)

Waterfall Cave, New South Wales, Australia	-35.42	150.11	604	N/A	Australia	2010	Direct radiocarbon dating on carbon particles within relict areas of pgiment	Direct	Exact Age	Yes	Painting	No	(4)
Wharton Hill, Australia	-32.64	139.77	41084*	41'084*	Australia	1992	Radiocarbon dating	Indirect	Minimum age	Unknown	Petroglyph	Yes	(57)
Winnemucca Lake, Nevada, Usa	40.12185	-119.339623	12'500	12'500	USA	2013	Radiocarbon dating	Indirect	Minimum age	Yes	Painting	No	(58)
Wonderwerk Cave, South Africa	-27.84673	23.55418	11'898*	1'138*	South Africa	1981	Radicarbon dating (minimum age)	Indirect	Minimum age	Unknown	Engraving	No	(59)
Wurik (Mann River E1002, 1004, 1005)., Northern Territory, Australia/Yikarrakkal (Mann River A1005), Northern Territory, Australia	-12.219481	134.143519	646	183	Australia	2010	Direct radiocarbon	Direct	Exact Age	Yes	Beeswax	Yes	(4)
Yam Camp, Cape York, Queensland, Australia	-15.7823	144.2341	682	602	Australia	2010	Direct radiocarbon dating from plant fibre binders (Earliest age) & indirect radiocarbon dating with oxalate sample (Latest age)	Direct	Exact Age, but latest date is minimum age	Yes	Painting	Yes	(4)
Yiwarlarlay, Northern Territory, Australia	-12.36686	130.88125	4'592	3'386	Australia	2010	Indirect radiocarbon	Indirect	Minimum age	Yes	Engraving	Yes	(4)
Yunta Springs, South Australia	-32.54	139.55	17'020	1'398	Australia	2010	Indirect radiocarbon	Indirect	Minimum age	Yes	Engraving	Mixed	(4)

Note: Dates marked with an *were calibrated by the authors, based on uncalibrated dates in the original article, or on date inferred as uncalibrated (see methods)

References for SI Table 1

1. White R, Mensan R, Bourrillon R, Cretin C, Higham TF, Clark AE, et al. Context and dating of Aurignacian vulvar representations from Abri Castanet, France. Proc Natl Acad Sci. 2012;109: 8450–8455.
2. García-Diez M, Hoffmann DL, Zilhão J, de las Heras C, Lasheras JA, Montes R, et al. Uranium series dating reveals a long sequence of rock art at Altamira Cave (Santillana del Mar, Cantabria). J Archaeol Sci. 2013;40: 4098–4106.
3. González-Sainz C, Ruiz-Redondo A, Garate-Maidagan D, Iriarte-Avilés E. Not only Chauvet: dating Aurignacian rock art in Altxerri B Cave (northern Spain). J Hum Evol. 2013;65: 457–464.
4. Langley MC, Taçon PSC. The age of Australian rock art: A review. Aust Archaeol. 2010;71: 70–73. doi:10.1080/03122417.2010.11689386
5. Steelman KL, Ramírez FC, Valcarce RF, Guilderson T, Rowe MW. Direct radiocarbon dating of megalithic paints from north-west Iberia. Antiquity. 2005;79: 379–389.
6. Thackeray AI. Dating the rock art of southern Africa. Goodwin Ser. 1983;4: 21–26. doi:10.2307/3858098
7. Bednarik RG, Kumar G, Watchman A, Roberts RG. Preliminary results of the EIP Project. 2005. Available: http://ro.uow.edu.au/scipapers/3611/
8. Mazel AD, Watchman AL. Dating rock paintings in the uKhahlamba-Drakensberg and the Biggarsberg, KwaZulu-Natal, South Africa. South Afr Humanit. 2003;15: 59–73.
9. Francis JE, Loendorf LL, Dorn RI. AMS radiocarbon and cation-ratio dating of rock art in the Bighorn Basin of Wyoming and Montana. Am Antiq. 1993;58: 711–737.
10. Taçon PS, Boivin N, Petraglia M, Blinkhorn J, Chivas A, Roberts RG, et al. Mid-Holocene age obtained for nested diamond pattern petroglyph in the Billasurgam Cave complex, Kurnool District, southern India. J Archaeol Sci. 2013;40: 1787–1796.
11. Watchman A, Cole N. Accelerator radiocarbon dating of plant-fibre binders in rock paintings from northeastern Australia. Antiquity. 1993;67: 355–358. doi:10.1017/S0003598X00045415
12. Bendrey R. Review of Wadi Sura–The Cave of Beasts edited by Rudolph Kuper. Pastoralism. 2014;4: 2. doi:10.1186/2041-7136-4-2
13. Valladas H, Clottes J, Geneste J-M, Garcia MA, Arnold M, Cachier H, et al. Palaeolithic paintings: Evolution of prehistoric cave art. Nat Lond. 2001;413: 479. doi:http://dx.doi.org/10.1038/35097160
14. Pike AWG, Hoffmann DL, García-Diez M, Pettitt PB, Alcolea J, Balbín RD, et al. U-series dating of paleolithic art in 11 caves in Spain. Science. 2012;336: 1409–1413. doi:10.1126/science.1219957
15. Valladas H, Cachier H, al et. Direct radiocarbon dates for prehistoric paintings at the Altamira, El Castillo and Niaux caves. Nat Lond. 1992;357: 68.
16. Valladas H, Tisnérat-Laborde N, Cachier H, Arnold M, Quirós FB de, Cabrera-Valdés V, et al. Radiocarbon AMS Dates for paleolithic cave paintings. Radiocarbon. 2001;43: 977–986. doi:10.1017/S0033822200041643
17. Butzer KW, Fock GJ, Scott L, Stuckenrath R. Dating and context of rock engravings in southern Africa. Science. 1979;203: 1201–1214.
18. Zorich Z. From the Trenches - Drawing Paleolithic Romania - Archaeology Magazine Archive. [cited 20 Jul 2017]. Available: http://archive.archaeology.org/1201/trenches/coliboaia_cave_romania_charcoal_drawings.html
19. Huyge D, Watchman A, De Dapper M, Marchi E. Dating Egypt's oldest 'art': AMS 14C age determinations of rock varnishes covering petroglyphs at El-Hosh (Upper Egypt). Antiquity. 2001;75: 68–72.
20. Mercier N, Valladas H, Aubry T, Zilhão J, Jorons JL, Reyss J-L, et al. Fariseu: first confirmed open-air palaeolithic parietal art site in the Côa Valley (Portugal). Antiquity. 2006;80. Available: http://www.antiquity.ac.uk/projgall/mercier/
21. Sand C, Valladas H, Cachier H, Tisnérat-Laborde N, Arnold M, Bolé J, et al. Oceanic rock art: first direct dating of prehistoric stencils and paintings from New Caledonia (Southern Melanesia). Antiquity. 2006;80: 523–529.
22. Watchman A. A review of the theory and assumptions in the AMS dating of the Foz Côa petroglyphs, Portugal. Rock Art Res. 1996;13: 21–29.
23. Moure Romanillo A, Gonzalez Morales M. Datation 14C d'une zone décorée de la grotte Fuente del Salin. Int Newsl Rock Art - INORA. 1992.
24. Clottes J, Valladas H, Cachier H, Arnold M. Des dates pour Niaux et Gargas. Bull Société Préhistorique Fr. 1992;89: 270–274. doi:10.3406/bspf.1992.9532
25. Geib PR, Fairley HC. Radiocarbon Dating of Fremont Anthropomorphic Rock Art in Glen Canyon, South-central Utah. J Field Archaeol. 1992;19: 155–168. doi:10.1179/009346992791548932
26. Valladas H, Kaltnecker E, Quiles A, Tisnérat-Laborde N, Genty D, Arnold M, et al. Dating French and Spanish prehistoric decorated caves in their archaeological contexts. Radiocarbon. 2013;55: 1422–1431. doi:10.1017/S0033822200048359

27. Broglio A, De Stefani M, Gurioli F, Pallecchi P, Giachi G, Higham T, et al. L'art aurignacien dans la décoration de la Grotte de Fumane. L'Anthropologie. 2009;113: 753–761. doi:10.1016/j.anthro.2009.09.016
28. Ambert P, Guendon J-L, Galant P, Quinif Y, Gruneisen A, Colomer A, et al. Attribution des gravures paléolithiques de la grotte d'Aldène (Cesseras, Hérault) à l'Aurignacien par la datation des remplissages géologiques. /data/revues/16310683/00040003/04001691/. [cited 25 Jul 2017]. Available: http://www.em-consulte.com/en/article/30133
29. Jaubert J, Genty D, Valladas H, Camus H, Courtaud P, Ferrier C, et al. The chronology of human and animal presence in the decorated and sepulchral cave of Cussac (France). Quat Int. 2017;432: 5–24. doi:10.1016/j.quaint.2016.01.052
30. Plagnes V, Causse C, Fontugne M, Valladas H, Chazine J-M, Fage L-H. Cross dating (Th/U-14 C) of calcite covering prehistoric paintings in Borneo. Quat Res. 2003;60: 172–179.
31. Nelson DE, Chaloupka G, Chippindale C, Alderson MS, Southon JR. Radiocarbon dates for beeswax figures in the prehistoric rock art of Northern Australia. Archaeometry. 1995;37: 151–156. doi:10.1111/j.1475-4754.1995.tb00733.x
32. Steelman KL, Rowe MW, Shirokov VN, Southon JR. Radiocarbon dates for pictographs in Ignatievskaya Cave, Russia: Holocene age for supposed Pleistocene fauna. Antiquity. 2002;76: 341–348. doi:10.1017/S0003598X00090426
33. Taçon PS, Aubert M, Gang L, Decong Y, Hong L, May SK, et al. Uranium-series age estimates for rock art in southwest China. J Archaeol Sci. 2012;39: 492–499.
34. Stasack E, Dorn RI, Lee G. First direct 14C ages on Hawaiian petroglyphs. Asian Perspect. 1996;35: 51–72.
35. Shchelinsky VE. Some results of new investigations at the Kapova Cave in the southern Urals. Proc Prehist Soc. 1989;55: 181–191. doi:10.1017/S0079497X00005387
36. Neves WA, Araujo AGM, Bernardo DV, Kipnis R, Feathers JK. Rock art at the pleistocene/holocene boundary in eastern South America. Petraglia MD, editor. PLoS ONE. 2012;7: e32228. doi:10.1371/journal.pone.0032228
37. O'Connor S, Aplin K, Pierre ES, Feng Y. Faces of the ancestors revealed: discovery and dating of a Pleistocene-age petroglyph in Lene Hara Cave, East Timor. Antiquity. 2010;84: 649–665. doi:10.1017/S0003598X00100146
38. Russ J, Hyman M, Rowe M. Direct radiocarbon dating of rock art. Radiocarbon. 1992;34: 867–872.
39. di Lernia S, Gallinaro M. The date and context of neolithic rock art in the Sahara: engravings and ceremonial monuments from Messak Settafet (south-west Libya). Antiquity. 2010;84: 954–975.
40. Mazel AD, Watchman AL. Accelerator radiocarbon dating of Natal Drakensberg paintings: results and implications. Antiquity. 1997;71: 445–449.
41. David B, Barker B, Petchey F, Delannoy J-J, Geneste J-M, Rowe C, et al. A 28,000 year old excavated painted rock from Nawarla Gabarnmang, northern Australia. J Archaeol Sci. 2013;40: 2493–2501. doi:10.1016/j.jas.2012.08.015
42. Corchón Rodríguez MS, Valladas H, Bécares Pérez J, Arnold M, Tisnerat N, Cachier H. Datación de las pinturas y revisión del Arte Paleolítico de Cueva Palomera (Ojo Guareña, Burgos, España). 2009. Available: https://gredos.usal.es/jspui/handle/10366/70469
43. Thaw UA. The "neolithic" culture of the Padah-lin Caves. Asian Perspect. 1971;14: 123–133.
44. Roosevelt AC, Lima da Costa M, Lopes Machado C, Michab M, Mercier N, Valladas H, et al. Paleoindian cave dwellers in the Amazon: the peopling of the Americas. Sci-N Y THEN Wash-. 1996; 373–384.
45. Mulvaney K. Dating the Dreaming: extinct fauna in the petroglyphs of the Pilbara region, Western Australia. Archaeol Ocean. 2009;44: 40–48.
46. Sauvet G. À la recherche du temps perdu. Méthodes de datations en art préhistorique. Available: https://www.researchgate.net/profile/Georges_Sauvet2/publication/289376793_A_la_recherche_du_temps_perdu_methodes_de_datations_en_art_prehistorique_L'exemple_des_sites_aurignaciens/links/568c0e1f08ae197e42689524.pdf
47. Huyge D, Vandenberghe DA, De Dapper M, Mees F, Claes W, Darnell JC. First evidence of Pleistocene rock art in North Africa: securing the age of the Qurta petroglyphs (Egypt) through OSL dating. Antiquity. 2011;85: 1184–1193.
48. Pike AWG, Gilmour M, Pettitt P, Jacobi R, Ripoll S, Bahn P, et al. Verification of the age of the Palaeolithic cave art at Creswell Crags, UK. J Archaeol Sci. 32: 1649–1655.
49. Bonneau A, Brock F, Higham T, Pearce DG, Pollard AM. An improved pretreatment protocol for radiocarbon dating black pigments in San rock art. Radiocarbon. 2011;53: 419–428.
50. Cole N, Watchman A. AMS dating of rock art in the Laura Region, Cape York Peninsula, Australia–protocols and results of recent research. Antiquity. 2005;79: 661–678.

51. Fontugne M, Shao Q, Frank N, Thil F, Guidon N, Boeda E. Cross-Dating (Th/U-14 C) of Calcite Covering Prehistoric Paintings at Serra da Capivara National Park, Piaui, Brazil. Radiocarbon. 2013;55: 1191–1198.
52. Jerardino A, Swanepoel N. Painted Slabs from Steenbokfontein Cave: The Oldest Known Parietal Art in Southern Africa. Curr Anthropol. 1999;40: 542–547. doi:10.1086/200051
53. Aubert M, Brumm A, Ramli M, Sutikna T, Saptomo EW, Hakim B, et al. Pleistocene cave art from Sulawesi, Indonesia. Nature. 2014;514: 223.
54. Hachid M, Le Quellec J-L, Amara A, Beck L, Heddouche A, Kaltnecker E, et al. Quelques résultats du projet de datation directe et indirecte de l'art rupestre saharien. Signs Which Times. 2012; 71–96.
55. Simek JF, Cressler A, Herrmann NP, Sherwood SC. Sacred landscapes of the south-eastern USA: prehistoric rock and cave art in Tennessee. Antiquity. 2013;87: 430–446.
56. Bednarik RG. An overview of Asian palaeoart of the Pleistocene. Proceedings of the IFRAO Congress. 2010. Available: http://www.ifrao.com/wp-content/uploads/2015/08/12Asia2.pdf
57. Dorn RI, Clarkson PB, Nobbs MF, Loendorf LL, Whitley DS. New Approach to the Radiocarbon Dating of Rock Varnish, with Examples from Drylands. Ann Assoc Am Geogr. 1992;82: 136–151.
58. Benson LV, Hattori EM, Southon J, Aleck B. Dating North America's oldest petroglyphs, Winnemucca Lake subbasin, Nevada. J Archaeol Sci. 2013;40: 4466–4476.
59. Thackeray AI, Thackeray JF, Beaumont PB, Vogel JC. Dated rock engravings from Wonderwerk Cave, South Africa. Science. 1981;214: 64–67. doi:10.1126/science.214.4516.64
