# Supplementary material for: Stabilization of cultural innovations depends on population density: Testing an epidemiological model of cultural evolution against a global dataset of rock art sites and climate-based estimates of ancient population densities
Source: PLoS One. 2021 Mar 17;16(3):e0247973. doi: 10.1371/journal.pone.0247973 (PMC7968670; doi:10.1371/journal.pone.0247973)
Supplement: S1 Fig — Posterior distributions for the γ, ξ, and ε parameters for the epidemiological model as shown in Fig 3A (observed site detection rates, for the full archaeological dataset and the combined population estimates in [28,29]): A. posterior distribution for γ; B. posterior distribution for Ϛ; C. posterior distribution for ε. (PDF) [file pone.0247973.s003.pdf]

S1 Figure

Posterior parameter distributions for the epidemiological model

A B C

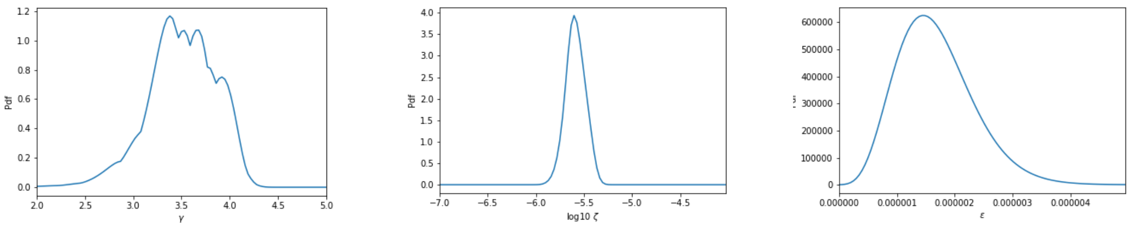

S1 Figure: Posterior distributions for the γ, ξ, and ε parameters for the epidemiological model as shown in Fig 3A (observed site detection rates, for the full archaeological dataset and the combined population estimates in [28,29]): A. posterior distribution for γ; B. posterior distribution for ζ; C. posterior distribution for ε.
