# Supplementary material for: Stabilization of cultural innovations depends on population density: Testing an epidemiological model of cultural evolution against a global dataset of rock art sites and climate-based estimates of ancient population densities
Source: PLoS One. 2021 Mar 17;16(3):e0247973. doi: 10.1371/journal.pone.0247973 (PMC7968670; doi:10.1371/journal.pone.0247973)
Supplement: S2 Fig — Cumulative frequency distribution for sites (blue) and non-sites (red) The inferred critical threshold (ρ∗) is shown in green. A: Eriksson; B: Australia; C: France-Spain-Portugal; D: Rest of the World; E: 0–9999 years ago; F: 10000–46300 years ago; G: Eriksson exact direct; H: Including sites with inferred population of zero; I: Timmermann. Frequency distributions for Eriksson j = 1.5, Eriksson j = 2.5 and Eriksson j = 6.0 are identical to the distributions in A and are not shown. (PDF) [file pone.0247973.s004.pdf]

S2 Figure

Cumulative Frequency Distributions for Sites and Non-Sites

A

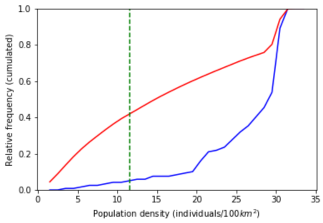

B

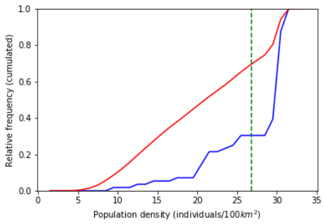

C

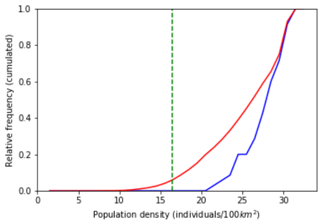

D

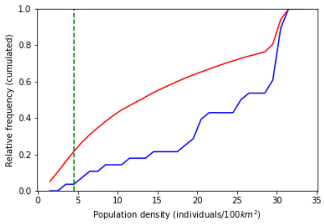

E

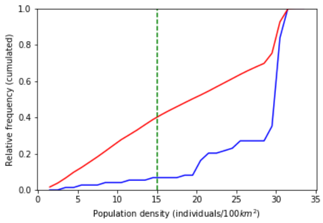

F

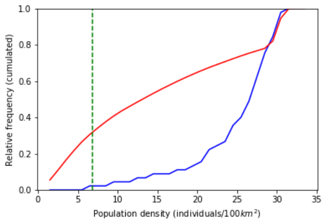

G

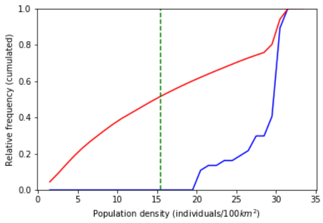

H

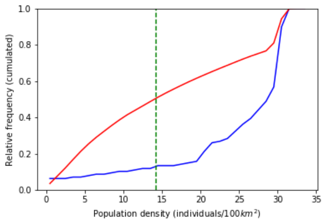

I

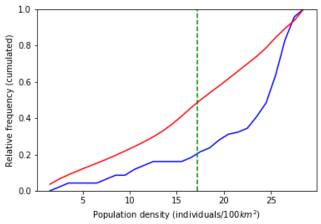

S2 Figure. Cumulative frequency distribution for sites (blue) and non-sites (red) The inferred critical threshold (ρ^*) is shown in green. A: Eriksson; B: Australia; C: France-Spain-Portugal; D: Rest of the World; E: 0-9999 years ago; F: 10000-46300 years ago; G: Eriksson exact direct; H: Including sites with inferred population of zero; I: Timmermann. Frequency distributions for Eriksson φ=1.5, Eriksson φ=2.5 and Eriksson φ=6.0 are identical to the distributions in A and are not shown.
