# Supplementary material for: Stabilization of cultural innovations depends on population density: Testing an epidemiological model of cultural evolution against a global dataset of rock art sites and climate-based estimates of ancient population densities
Source: PLoS One. 2021 Mar 17;16(3):e0247973. doi: 10.1371/journal.pone.0247973 (PMC7968670; doi:10.1371/journal.pone.0247973)
Supplement: Attachment — Submitted filename: Response to reviewers rw 200608.pdf [file pone.0247973.s005.pdf]

Geneva, June 8, 2020

Dear Editor,

Following our exchange of emails in February 2020, we are submitting a revised version of our manuscript PONE-D-19-31833. We thank you and your reviewers for the time and expertise they have dedicated to our work and for your flexibility in extending our submission deadline during the COVID-19 emergency. In this new version of our study, we attempt to resolve issues raised by reviewers, and more generally to clarify the intentions and significance of our work, as further detailed below.

Comments from Reviewer 1

"The term "globals" comes off initially as very strange and confused me for some time. Spend another sentence explaining that you are defining cells in your raster data set based on their intersection with your data set of archaeological sites. Adding "cells" (global cells, site cells) would also help disambiguate the terms "globals" and "sites."

We agree that our terminology here was not clear. We have thus replaced the term "globals" with the term "all cells". Additionally, we have included a clear definition of the concept of cell that was missing in our original text (see lines 89-91, 111-115

Change to "site dates" (proper adjectival form) or otherwise clarify grammar.

Done (see line 311)

The term in English is mobiliary art. Avoid unnecessary foreign-language terms.

We have decided to use the term "portable art" (see line 324)

"(…) you should expound a little bit more on how dates were handled. Did you use any calibrations/corrections (14C yrs BP versus cal BP presents a big difference)? How did you handle varying uncertainty terms? Or did you take all reported dates at face value?

(…) I see that not all of these 14C dates are calibrated. Comparing calibrated and uncalibrated dates, depending on what portion of the calibration curve you're dealing with, is like comparing apples and oranges. The best practice here would be to recalibrate all of the dates yourselves, or to exclude all uncalibrated dates. On the sort of very general temporal scale that you are dealing with, I don't think this necessitates rerunning your analysis. However it potentially causes serious issues with the chronological assignments of some of your sites that the reader should be made aware of."

Campus Biotech
Chemin des Mines 9
CH-1202 Geneva
Switzerland

Tel: +41 (0)21 693 7660
Email: info.bbp@epfl.ch
Web: bluebrain.epfl.ch
portal.bluebrain.epfl.ch

We thank your reviewer for emphasizing this issue. In the new version of our manuscript we have attempted to follow his suggestions.

When testing our model, we consistently used the we considered only the *oldest* date for each site. Where confidence intervals were given, we used the midpoint of the interval. oldest date recorded for a site. We reviewed the calibration status of all dates used in our analysis. Where original authors did not explicitly indicate the calibration status of radiocarbon dates, we attempted to infer their calibration status from the surrounding text and from the CIs for the dates. We then calibrated the uncalibrated dates, using Calib 7.0 [41] with the IntCal 13 calibration curve. Dates we recalibrated are indicated with an asterisk in SM Table 1. We explain the way we have handled dates in lines 334-341.

We recognize that the procedure used to infer the calibration status of radiocarbon dates when this was not explicitly indicated by original authors is prone to errors. To test the sensitivity of our results to these and other potential errors in dating, we performed a new analysis in which we excluded all sites without an exact age, obtained with direct methods and excluding radiocarbon dates that the original authors had not explicitly declared to be calibrated. The results of the analysis with this restricted dataset, (see Table 1) were very similar to those obtained with the full dataset. We describe the new study in 166-186 and 430-442.

This is a long and confusing sentence. Please clarify.

We agree with your reviewer and have rewritten the sentence (see lines 359-362)

"Can you be more specific? North American? Laurentide Ice Sheet? The Americas are a big place"

In our analysis, the original population density estimates for the North and South American continents contained in Eriksson 2012, were completely replaced by revised estimates from Raghaven, 2015 (see references). My co-first author, Anders Eriksson, was an author on both papers. The reasons for the replacement are explained in lines 379-384 of the manuscript

Reviewer 2

Reviewer 2's negative evaluation may be partially due to the "diffusionist" language we use to describe and interpret our model. To avoid misunderstanding, we have changed the title of our paper, and clarified the description and interpretation of our model. In what follows we respond to Reviewer 2's evaluation in greater detail.

The paper is not technically sound. The statistical analysis was not performed appropriately or rigorously

Reviewer 2 states that our work is not technically sound and denies that our statistical analysis was performed appropriately or rigorously. Unfortunately, he does not motivate these evaluations. His comments raise no issues about our sampling methods, rock art data, choice of controls, previously published population estimates, sample

size, checks for confounders, or use of different datasets to test our model. It is thus difficult for us to respond.

Reviewer 2's main argument is his skepticism that

> *"... such an approach can work for anything but the most basic utilitarian cultural traits because the size of population needed to borrow more complex aspects of symbolic culture need not extend beyond family level as ethnographic examples from Khoisan and Australian Aboriginal groups have shown (see exchange research by Polly Wiessner in Kalahari as an example)."*

He goes on to explain that

> *(...) the reason that rock art cannot serve as a test for this model is twofold: 1) there is insufficient rock art dated to know exactly when rock art appeared in any specific area; 2) our best guess at present, based on the partial data available, is that rock art arrived as part of the colonising repertoire of cultural traits in all continents except the place where it was invented: Africa. This means that rock art did not spread by a diffusionary process as is required by this model, but was brought as an embedded piece of cultural practice, hence, it cannot be used for the purposes employed here*

These objections appear to be based on misunderstandings of our model that we will discuss below. More importantly, they represent nothing more than the reviewer's personal opinion. Even if this opinion were widely shared, such a consensus would not be a valid reason to refuse engagement with our data and analytical results.

<u>The data we present provide overwhelming support for a relationship between population density and the presence of rock art, and strong support for the specific predictions of our model</u>. In these circumstances, a critical reviewer would normally seek to attack the results (e.g. by demonstrating systematic biases or cherry-picking of data), the analysis (e.g. by criticizing the choice of statistical methods), or the interpretation of the results (e.g. by suggesting alternative explanations for empirical findings). Reviewer 2 does not offer this kind of critique.

Unsubstantiated insinuation

Reviewer 2 writes

> *"I question why these [other technologies] were not tried in preference to rock art where the extent and nature of our dating is notoriously problematic? **My guess is that these authors must have tried these other cultural traits first and failed.** (our emphasis)"*

We would like to state emphatically that we have not hidden any negative results from previous studies (the file drawer effect). It is not acceptable that this sort of unsubstantiated insinuation should be "thrown in" in the middle of the review report.

Diffusionism

We recognize that our use of "diffusionist" language in the original version of our manuscript is liable to create misunderstandings. In reality, we do not make any claim about the history of rock art, globally or in any particular region. More specifically, we never claim or assume that the global distribution of rock art is the result of a process of diffusion between populations (classical diffusionism). What we actually model is the process allowing a cultural innovation to be stably maintained (i.e. to become endemic) within a metapopulation of communicating subpopulations. We have now made this point explicit in the title of our study, the abstract, and the text describing our model (see lines 57-63).

Our underlying assumption is that the ability to produce rock art, like other cultural traits, is transmitted from person to person and from subpopulation to subpopulation. Reviewer 2 posits that "*rock art arrived as part of the colonising repertoire of cultural traits in all continents except the place where it was invented*", If this was so, the ability to create rock art was necessarily transmitted vertically from generation to generation and horizontally between subpopulations, over very long periods of time. Furthermore, transmission must have been sufficiently reliable to ensure that the trait was never completely lost. In brief, transmission was vital. This is true regardless of the complexity or otherwise of the trait in question.

The model we present in our manuscript, and other demographic models of cultural evolution (see references 10-16 in our paper), propose that successful transmission over many generations depends on demographics. It is possible that small social groups, with intense social contact between group members, and high local population density could maintain complex traits (e.g. rock art), even when the mean population density in the territory they inhabit is low. This is what appears to have occurred in the ethnographic examples cited by reviewer 2. However, small groups are extremely vulnerable to stochastic events in their environment and are unlikely to survive over long periods of time (see references 12-13 in our manuscript). If this is so, most such complex traits will disappear without leaving a trace in the archaeological record. In our manuscript we argue that the traits most likely to leave such traces are those that spread between subpopulations, ensuring that the cultural trait survives even when individual subpopulations are destroyed, disintegrate or lose their skills. This in turn requires a minimum level of inter-group contact. This is the reason, we argue, why most of the rock art sites in our dataset are in territories with population density beyond the critical threshold.

The need for exact dating of rock art sites

Reviewer 2 believes that our analysis depends on exact knowledge of the dates at which rock art first appeared in a specific location. This is a misunderstanding. In reality, the only requirement for our analysis is an unbiased sample of rock art sites with accurate dates. As reported in our Methods section, we use the *oldest available* dates for each site (not the date for the *first actual appearance* of art at the site which is unknown and unknowable). This somewhat arbitrary decision was driven by technical considerations.

Many excavations yield different samples of rock art with different dates. Since these cannot be treated as independent data points, we decided to use a single point for each site. We did not choose the most recent date, because in many American and Australian sites the most recent findings are modern.

Finally, we note that reviewer 2 is mistaken about the availability of reliable dates for rock art sites. Many early reports were indeed very inaccurate. However the last thirty years have seen important advances in dating methodology. In particular, artifacts produced using organic materials can now be dated directly using radiocarbon methods. Many of the reports covered by our literature survey use such methods. In a control study, presented in our paper, we repeat our analysis, restricting our rock art data to sites where exact dates had been determined using direct methods and where all radiocarbon dates have been calibrated by the original authors. We find that the likelihood of our model given this "purified" data set is higher than with the data used in our original analysis (see lines 166-186, 430-442).

We hope that in view of these considerations and of the substantial revisions we have made to our manuscript, you will be able to accept our work for publication in PLOSONE.

Yours sincerely

Richard Walker

(on behalf of the other authors)
