# Supplementary material for: Stabilization of cultural innovations depends on population density: Testing an epidemiological model of cultural evolution against a global dataset of rock art sites and climate-based estimates of ancient population densities
Source: PLoS One. 2021 Mar 17;16(3):e0247973. doi: 10.1371/journal.pone.0247973 (PMC7968670; doi:10.1371/journal.pone.0247973)
Supplement: Attachment — Submitted filename: response to reviewers.docx [file pone.0247973.s006.docx]

Blue Brain Project

EPFL Campus Biotech

9 Chemin des Mines,

01202 Geneva

Switzerland

December 16, 2020

Dear Mr Lozano,

We would like to thank you and your reviewers for giving us the opportunity to resubmit our manuscript entitled “Stabilization of cultural innovations depends on population density: testing an epidemiological model of cultural evolution against a global dataset of rock art sites and climate-based estimates of ancient population densities”.

We are especially grateful to the reviewers for their thorough, careful and well-informed reading of our manuscript. Several of the comments challenged our initial assumptions, leading us to perform additional analyses, not included in the original manuscript, and to revise parts of the manuscript, where our original text was not sufficiently clear. We hope this new version of the manuscript does justice to their work. In what follows, we address comments, one by one, indicating in each case, any changes we have made to the manuscript. In each case, we discuss substantial remarks first, leaving less important issues to the end

**Reviewer 3**

1) **I don’t understand what is correlated with what. Was a single population size associated with each geographic cell (e.g. the highest population size estimate for that region over the past 46KY, or for the period 46ky-10ky, and then a separate size for 10ky to the present)?**

We apologize if our original text did not make this sufficiently clear. The population densities in our analysis represents the estimated population density of a hexagonal “cell” *for a specific 25 year time window*. Cells where the first recorded appearance of rock art falls within the time window for the cell are defined as “sites”. The “detection ratios” used in the analysis are computed by dividing the number of sites in a bin by the total number of non-sites (measured across the complete period covered by the analysis) . We have modified the presentation of the model (lines **87-92)** and our data analysis (lines **119-125**) to clarify our methods.

2) **... if things are done this way, and there are multiple dates for the same rock-art site spanning more than one 1000-year window, each of these dates should be considered, not only the oldest one.**

Excavations at some of the sites in our dataset have produced large numbers of rock art specimens, sometimes spanning thousands of years. If we had included one date (and associated population density) per specimen (instead of one per site), we would have (i) over-represented sites with large numbers of specimens (ii) included a large number of non-independent data points (estimated population densities at different times). There is a serious risk that treating datapoints from the same site as independent would *overestimate* empirical support for our model. We therefore prefer to maintain the more conservative approach described in our manuscript. We have provided additional text (lines **123-127)** motivating this design.

3) **...each site of rock paintings is something produced within a single generation, or maybe 5-10 generations (...) but it is then correlated with pop-size estimates whose time-resolution differs by an order of magnitude (probably thousands of years?**

This objection seems to derive from the misunderstanding discussed under point 1. We agree that the parameter of interest for our model is the rate of transmission over ecological time-scales. The size of the time window in our analysis (25y) matches this time-scale.

4) **...the data is super-sparse, so even if the model is/were the precise description of what actually happened in the world, there's no chance that you'd have the events in the dataset to support it.**

We believe that a sparse sample can be informative, even if small, on two conditions:

a. The phenomenon under investigation has a sufficiently large effect
b. The sample is representative of the population i.e. the sample is not systematically biased

As concerns the first condition, our revised manuscript includes new analyses documenting the proportions of sites and non-sites with sub-threshold population densities (see **Table 1)** and new graphs showing their relevant frequency distributions (see **SI Fig 2).** In our main analysis for example, we show that just 6/119 (0.50%) sites have population densities below the inferred critical threshold, compared to 42% of non-sites. The size of the observed effect makes it extremely unlikely that our results are due to noise in the data. The latest version of our software allows users to replicate these studies for themselves.

5) **These locations (our note: locations with large ancient population densities) are where archaeologists would have looked for such art as well, so there's a huge confirmation bias here....**

Your reviewer points to the possibility of huge bias in the archeological record of rock. We agree with this assessment and have changed the position of the relevant section in our manuscript to emphasize the importance of this issue We have also expanded and rewritten our discussion, including a new analysis to test the role of regional bias(see lines **199-235).**

The original manuscript already included analyses for the *two regions* in the world *(*France-Spain-Portugal, Australia) with the highest volume of attested rock art and two distinct periods (from 0-9,999 years ago, and from 10,000-46,300 years ago). These analyses show that the predicted effect of population density holds *within* as well as *between* regions and periods*.* We have now included an additional analysis using data for the remaining regions of the world. All five analyses show a strong relationship between the frequency of rock art and population density and in three out of five cases the epidemiological model is better supported than the proportional model (see lines **219-225** and the results in Table 1**).** The latest version of our software allows users to replicate these analyses.

To summarize, we have made a great effort to correct for the unavoidable biases present in our dataset, we believe with some success. Nonetheless, we agree with your reviewer that no correction can compensate for missing or biased data. The ultimate assessment of our model depends on results from future research. New text in the abstract (section on bias ( **26-27**), in the section on bias ( **228-234**) and in the conclusions (**302-304**) draws attention to this caveat.

6) **I don't think the finding that the epidemiological model gets more support than the simpler correlation-between-art-and-pop-size is more than an artifact of the way that the comparison was done. That is, without fully understanding the Bayesian-based model-selection that you did, I'm quite certain that in the bottom line the comparison favored the more complex model because it has more levels of freedom.**

To clarify our procedures we have added a paragraph to the methods section, describing our Bayesian model selection procedure ( **490-506**). We have also revised our text to show where the predictions of the epidemiological model coincide with those of the proportional model and where they diverge (**151-174; 219-228; 293-305)**

As concerns the substance of your reviewer’s objections, we still believe that there is strong evidence in favor of the epidemiological model.

a. In all our analyses we compute Bayes Factors for the epidemiological model vs. the proportional and null models. In our main analysis and in three out of five of our partial analyses, the epidemiological model is many orders of magnitude more likely than alternative models.
b. Our analyses show very few sites in cells with population densities below the inferred critical threshold Under the null hypothesis we would expect the proportion of sites with populations in this range to be approximately proportional to the proportion of cells. Even allowing for some dependencies in the data, the probability that the observed distribution could have arisen by chance is vanishingly small.
c. The proportional model predicts that site frequency will increase in direct proportion to population density. In our observations there is no measurable increase before population density reaches the critical threshold **(see SM fig 2)** . This is further evidence in favor of the epidemiological model.
d. The median population density for rock art sites is in the same high range (between 25 and 30 inhabitants/100 km^2^ ) in all our analyses. This supports the notion that rock art occurs not just where there are more people, but where there a lot of people (in historical terms).
e. As concerns the issue of complexity, the revised version of our manuscript formally accounts for differences using the Aikake Information Criterion (ref. **37**) - a measure of fit that penalizes models with a higher number of parameters. The results, included in Table **1** and described in lines **164-166**, **512-515,** shows lower values (better performance) for the Epidemiological model than for the proportional or null models. This is evidence that our results are not due to the complexity of the model.

**11) Excluding the sites in which cave art is found despite estimates of nearly zero population seems like cherry-picking; these are the strongest cases that would support a claim that there's no connection between density and presence of rock art. I don't understand the justification for throwing these out.**

We thank your reviewer for raising this potentially serious objection to our analysis.

Our original intention in excluding “uninhabited” cells from our analysis was to avoid the facile conclusion that cells with no inhabitants produce no rock art. However, we had not considered the possibility that our population model would identify some of our sites as uninhabited at the relevant dates.

To test the impact of excluding these sites, we have performed a new analysis which included sites and non-sites with inferred population densities of zero (. The analysis (supported in the latest version of our software) showed (to our relief!!!) that support for the epidemiological model is extremely strong *even after the “uninhabited” sites are included.* This result reflects the huge gap between the observed number of sites and the number expected under the null model. Even after inclusion of 8 additional sites, the gap remains extremely large. The need for this analysis and the results are described in lines **258-265**

**12)	In many (most?) SIR models, once an individual is in the Recovered/Removed category, they can't be re-infected (because of adaptive immunity or because of death; for transmission, the two are the same). I didn't see a mention of how/whether this is treated here. Worth mentioning.**

We have added additional text (lines **70-74)** to the original text, interpreting the meaning of “recovery” in the context of the model

**13)	One of the model parameters is the rate of recovery (loss of the cultural trait of painting on rocks). Wouldn't the straight-forward way to estimate this parameter be to use the distribution of date inferences for art from the same cell? Currently it seems that you excluded from your analysis all the later dates except the oldest one?**

Unfortunately, we do not believe it is possible to estimate the “rate of recovery” using the method suggested by your reviewer, for two reasons:

a. The parameter of interest for our model is the loss of “rock art” related skills in a specific population, measured on an ecologically realistic timescale (tens or hundreds of years). However, populations are mobile. Thus disappearance of rock art at a site does not necessarily represent the loss of the relevant cultural traits from the population - the population may simply have moved. Conversely, the presence of rock art over a long period of time does not necessarily imply continued rock art production by a single population - it may reflect production by multiple populations occupying the site in sequence.
b. Given the fragmentation of the archaeological record, it is difficult to determine if the disappearance of rock art after a certain date is due to the loss of relevant cultural traits or to accidents of ecology, preservation and excavation.

In view of these considerations, we prefer to estimate the recovery rate using the methods described in our manuscript.

**Final comments**

**The bottom line is that I still think it's worth publication, but that the limitations need to be discussed and highlighted early on. See my comments below and summary at the end.**

Your reviewer is correct that the available rock-art data is sparse and that estimates of ancient population densities are necessarily crude. And, as he/she points out, the archaeological record is almost certainly biased. The revised version of our manuscript includes new analyses designed to give a clearer picture of these issues. However, we fully accept that no analysis can completely obviate the problems indicated by your reviewer. As mentioned earlier, we have revised the abstract**,** the discussion of bias and the conclusions **,** to recognize this reality.

**Reviewer 4**

…. **the authors ...tried to run the experiment with different relations between group interaction and population density (introducing a parameter $\phi$ to generalise their model). They say the results are the same but also that they can't show the results. Would it be possible to have them at some point? recalculating the Table 1 for, let say, 3 different values of $\phi$ and add that to the SI should not be that hard or I am wrong?**

We agree with your reviewer that it is useful to show the results from this computation, which are now included in the last three columns of **Table 1**. We tested the model for phi=1.5, phi=2.5 and phi=6 (an extreme value). Choosing different values for phi slightly changes the most likely value for the critical threshold. However this is strongly constrained by the data and the changes are consequently small. Although the original model with phi=2 fits the data slightly better than the alternative models, the differences are insufficient to draw firm conclusions about the most likely value of phi. We have added text t (lines **284 - 289)** illustrating these results.

**Following on this interaction part of the epidemiological model, the authors also decide to "normalized" the parameter $\beta$ to one. This sounds like a strong move to me, as if I understood correctly, this is the parameter representing the "virality" of new cultural variants, _ie_ the rate at which a cultural traits it's shared between populations. Thus, one would expect than with higher $\beta$, the need for denser population will decreases.**

Your reviewer is correct that that beta is an indicator of virality or “infectivity” and that the higher its value the lower is the need for denser populations. The implication is that easily transmitted innovations (e.g., innovations that are relatively simple) can become endemic at lower population densities than those that are less infective (e.g. innovations that are more complex)

**Then, one could argue that by setting $\beta$ this way the authors are forcing the model to work only with denser populations, while other path to CCE may be possible. ...the authors should spend a few more words to justify there choice here (maybe explaining why they think this won't change the overall outcome of the model if they think so? :**

The model testing procedure described in our paper determines a single value for the critical level of population density beyond which an innovation becomes endemic.

Because the model predictions depend only on $\rho^{*}$, it is not possible to determine the individual values of $\alpha$, $\beta$ and $\gamma$ but only the value of $\rho^{*}$ . In an analysis where $\rho^{*}$is defined to take a single value, curve fitting will automatically compensate for changes in the value of $\beta$by changing the values of $\gamma$and $\alpha.$ As a consequent normalization of the value of $\beta$ to 1, has no impact on the value of the critical population density.

On a side note, the value of beta is highly relevant for analyses where $\rho^{*}$ can assume multiple values - e.g., analyses comparing transmission dynamics for different classes of cultural innovation

.

**It seems that the Figure 1 wasn't included with the publication...**

We apologize for this omission. Figure 1 is now included.

**I understood that Figure 3B (and some elements of 3A) shows the values for the "threshold", which is called $\rho^{*}$ throughout the main text, but it's reported as $r^{*}$ in this figure. I may be wrong and $r^{*}$ is something else, but then I was not able to find what it was referring to. Also, I found the left panel of this figure confusing (the A panel) :**

Our revised manuscript includes a new version of Figure 3, where the threshold is correctly labeled. We have also rearranged Panel A, hopefully making it easier to read, and including the posterior distribution for the threshold.

**I have some questions regarding the model and the Bayesian inference. it would be nice to have an idea of those values, maybe a table summarising all parameters inferred, with a brief description of what they mean and their prior distribution**

As suggested by your reviewer, our revised manuscript now includes a Table (SI Table 2) showing the priors used for parameter estimation. The posterior distribution for $\rhos^{*}$ is shown in Figure 3A**.** Unfortunately we were unable to devise a helpful representation of the joint posterior probabilities for the three parameters in our model.

**It would help also to have a few sentences explaining how the likelihood is computed.**

In response to this, and a similar request from reviewer 3, we have added a short paragraph describing the details of the computation (lines **489-502**).

**... minor problems popped up when I first tried to use the python code from the git repository (typos in folders' name and necessary packages that were not mentioned in the readme.txt)**

We have released a new version of the software where these problems are hopefully corrected. We have also corrected some of the variable names to align them with the terminology used in the paper. The new release allows users to reproduce all the new analyses included in the revised version of our manuscript. It is freely available, as of today, at *https://github.com/rwalker1501/cultural-epidemiology.git.*

**Minor typos**

We have corrected our reference "Qitdlarssuaq: L’histoire d’une migration polaire". It appears that our reference manager cannot read French. Unfortunately we were not able to find the problem with the parentheses in line 150.

**Other corrections**

On rereading our manuscript and performing the new analyses requested by your reviewers we found two issues where the original version was unclear or inconsistent.

1) The manuscript (and the associated software) were not always consistent in the nomenclature for cells. In the previous version of our manuscript we compare the frequency distribution of sites against the frequency distribution of “all cells”. In reality we were comparing it against the distribution of “non-sites”. In the new version of the manuscript we make this explicit. For practical purposes, the two distributions are indistinguishable. However, the new terminology is more accurate formally, and, we think, clearer for the reader.
2) In our previous analysis we were not consistent in the labelling and definition of the geographical area previously referred to as “France-Spain”. We have now expanded the definition to formally include sites located in Portugal and repeated the analysis on this basis.

Hoping that you and your reviewers will appreciate this revised version of our manuscript

Yours sincerely

Richard Walker

(corresponding author)
