# Supplementary material for: Stabilization of cultural innovations depends on population density: Testing an epidemiological model of cultural evolution against a global dataset of rock art sites and climate-based estimates of ancient population densities
Source: PLoS One. 2021 Mar 17;16(3):e0247973. doi: 10.1371/journal.pone.0247973 (PMC7968670; doi:10.1371/journal.pone.0247973)
Supplement: Attachment — Submitted filename: response to reviewers.docx [file pone.0247973.s007.docx]

Blue Brain Project

EPFL Campus Biotech

9 Chemin des Mines,

01202 Geneva

Switzerland

February 16, 2021

Dear Dr Lozano,

We would like to thank you and your reviewers for their positive assessment of our manuscript.

In answer to the query from Reviewer 4, we have extended our discussion of the results we obtained by varying the value of  $\phi$ in our generalized model (see lines 262-286). On the basis of our results, we suggest that it is unlikely that high population mobility can *completely* compensate the negative effects of low population density, at least in the case of rock art. It is nonetheless likely that high mobility and long-distance social contacts can compensate for *some* of these effects, creating Culturally Effective Populations that are much larger than the census population of a specific area, as suggested in reference 19.

In reply to a useful suggestion from the editor, we have tried to eliminate a number of redundancies between the Introduction, the Methods and the Results. To this end, we have eliminated the description of the model from the Methods, moved the formal derivation of the model to Supporting Information, and introduced a concise description of the model immediately after the Introduction. As in the previous version, we have placed the Methods at the end of the article. We believe that this approach will make the article more accessible for non-specialist readers.

Hoping that you and your reviewers will appreciate these changes and accept our manuscript for publication in PLOS ONE-

Yours sincerely

Richard Walker

(corresponding author)
